# Immunoglobulin G4-Related Disease Accompanied by Primary Myelofibrosis: Case Report

**DOI:** 10.3389/fmed.2021.638794

**Published:** 2021-05-04

**Authors:** Ziwei Liu, Shangzhu Zhang, Wen Zhang, Jun Feng, Mengtao Li, Xiaofeng Zeng

**Affiliations:** ^1^Department of Rheumatology, Peking Union Medical College Hospital, Peking Union Medical College and Chinese Academy of Medical Science, Beijing, China; ^2^Department of Hematology, Peking Union Medical College Hospital, Peking Union Medical College and Chinese Academy of Medical Science, Beijing, China

**Keywords:** immunoglobulin G4–related disease, primary myelofibrosis, acute myeloid leukemia, lymphadenopathy, myeloproliferative neoplasm

## Abstract

Immunoglobulin G4-related disease (IgG4-RD) is a heterogeneous autoimmune fibrosing disorder that presents common pathologic features but with unclear etiology. We report a rare case of IgG4-RD accompanied by primary myelofibrosis that eventually transformed into acute myeloid leukemia. A 50-year-old woman suffered from progressive lacrimal and parotid gland enlargement, diaphoresis, and rapid weight loss. Important clinical findings included remarkable leukocytosis, hyperglobulinemia, and splenomegaly. IgG4-RD was confirmed by salivary gland biopsy. Meanwhile, myelofibrosis was diagnosed according to histopathological findings of bone marrow and genetic mutation test of peripheral blood. The patient was on corticosteroid treatment. However, she developed into acute myeloid leukemia (AML) in the 8th month of follow-up. Our case suggested that myeloproliferative neoplasm (MPN) may co-occur with IgG4-RD. Bone morrow aspiration and genetic tests are helpful for throughout evaluation. An active search for hematological malignancies is warranted at diagnosis and during follow-up for patients who present with unexplained leukocytosis, pancytopenia, splenomegaly, or weight loss.

## Introduction

Immunoglobulin G4-related disease (IgG4-RD) is an immune-mediated fibroinflammatory disease that can involve multiple organ systems. Due to a heterogeneity in clinical features, intensive investigation is often required for differential diagnosis. Recently, an increased incidence of solid malignancies was found in patients with IgG4-RD (1, 2). Hematological malignancies, mainly B-cell lymphoma, were relatively less reported (3). We present the first reported association of IgG4-RD and myelofibrosis with eventual acute myeloid leukemia (AML) transformation. Future studies are required to understand the potential relationship of these two distinct disease spectrums.

## Case Report

**Figure 1:**
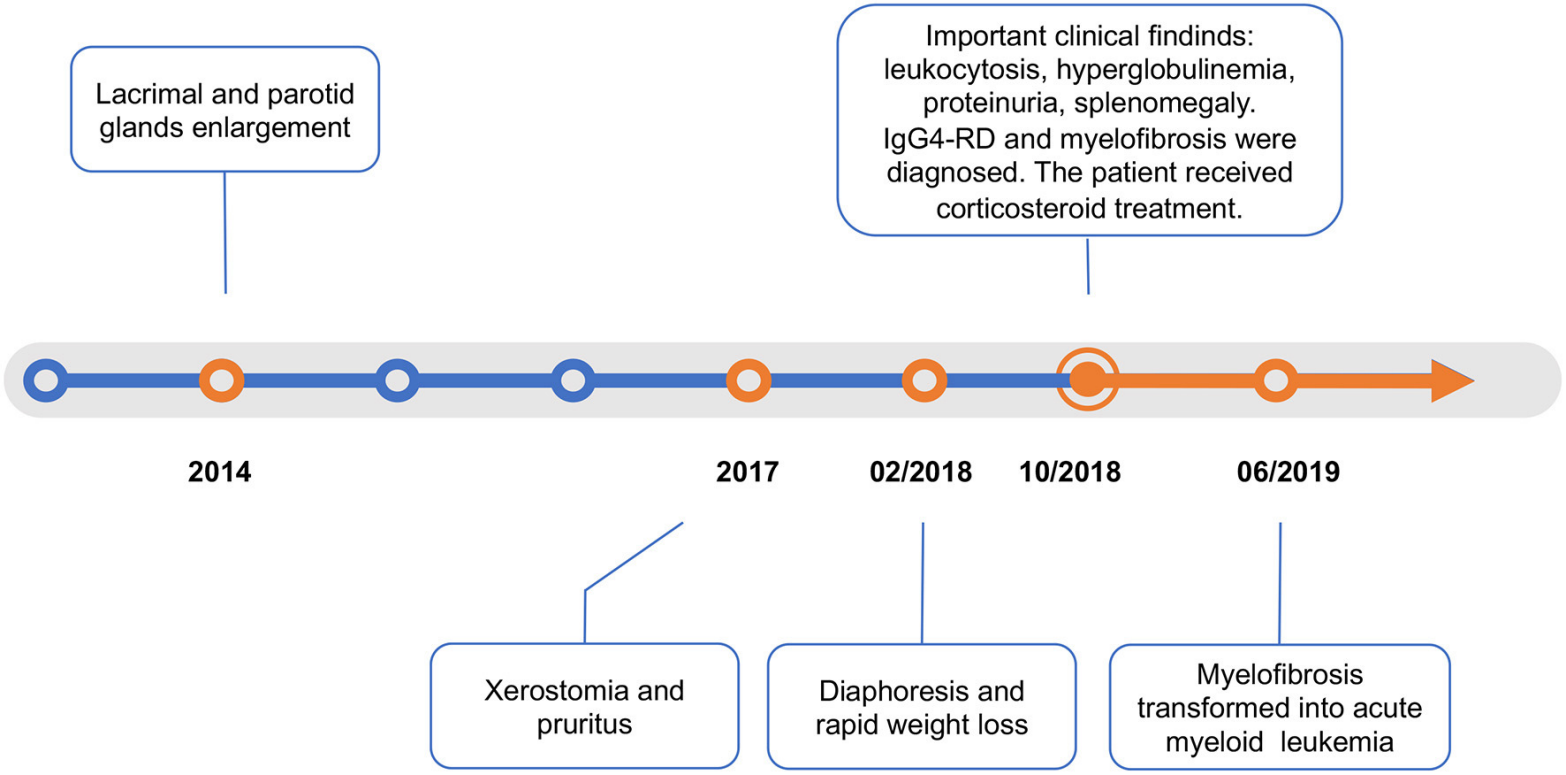
Timeline.

A 50-year-old woman was referred to the rheumatology department in October 2018. She reported a 4-year history of lacrimal and parotid gland enlargement that progressed gradually without treatment (Figure 1). Approximately 2 years before presentation, xerostomia and pruritus developed. The patient used to take traditional Chinese herbs but ceased shortly afterwards since the symptoms were not alleviated. Eight months before presentation, she started to suffer from fatigue, excessive diaphoresis, and rapid weight loss. She reported no rash, fever, or arthritis. The patient had a history of seasonal anaphylactic rhinitis but did not take any medication. She worked as an administrative staff at a local college and had a healthy daughter. There was no family history of autoimmune or malignant diseases.

**Figure 2:**
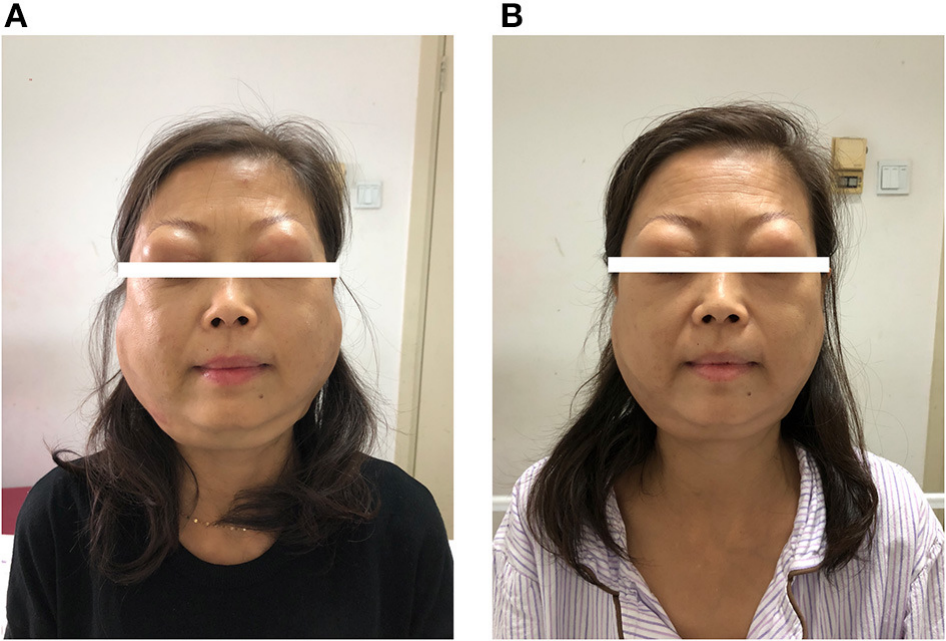
**(A)** On admission. **(B)** Two weeks after treatment.

On physical examination, the patient appeared to be anemic. Bilateral lacrimal and parotid glands expanded substantially (Figure 2A). Splenomegaly was found as the inferior margin of the spleen was palpated lower than the anterior superior iliac spine. She also had diffuse superficial lymphadenopathy.

Lab tests reported a remarkably elevated white blood cell count of 450,000 per cubic millimeter. Hemoglobin was 81 g/l. Bilirubin and pancreatic enzymes were normal. Serum immunoglobulin G (IgG) was 83 g/l, and IgG4 was 2,410 mg/dl on protein electrophoresis. Hypocomplementemia was presented with C3 0.249 g/l and C4 0.009 g/l. For rheumatic evaluation, antinuclear antibodies and anti-neutrophil cytoplasmic antibodies (ANCA) were negative. The serum creatinine was normal. The 24-h urine protein was 1.82 g. Renal perfusion imaging reported right hydronephrosis and decreased function on both kidneys.

**Figure 3:**
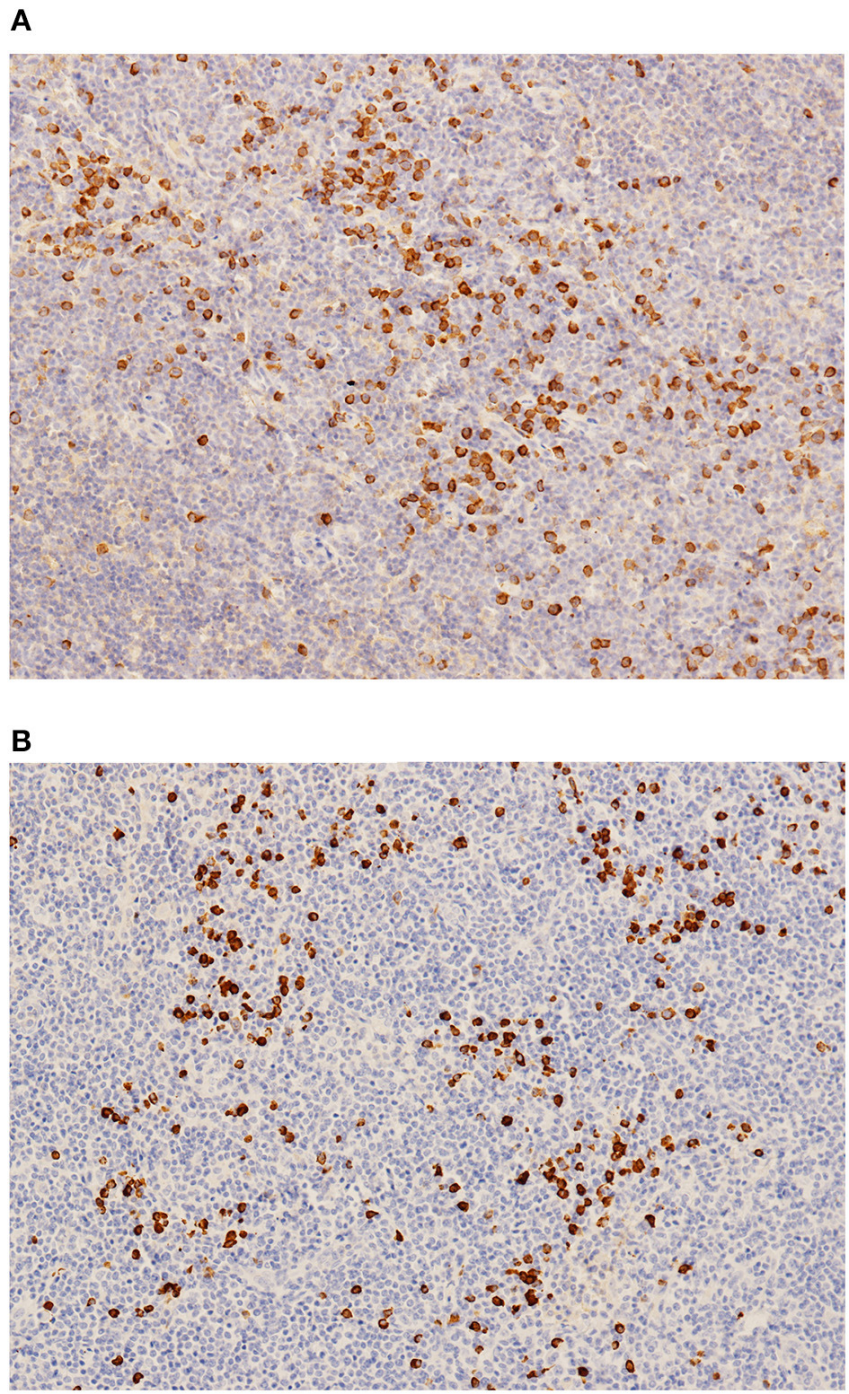
**(A)** IgG staining. **(B)** IgG4 staining.

**Figure 4:**
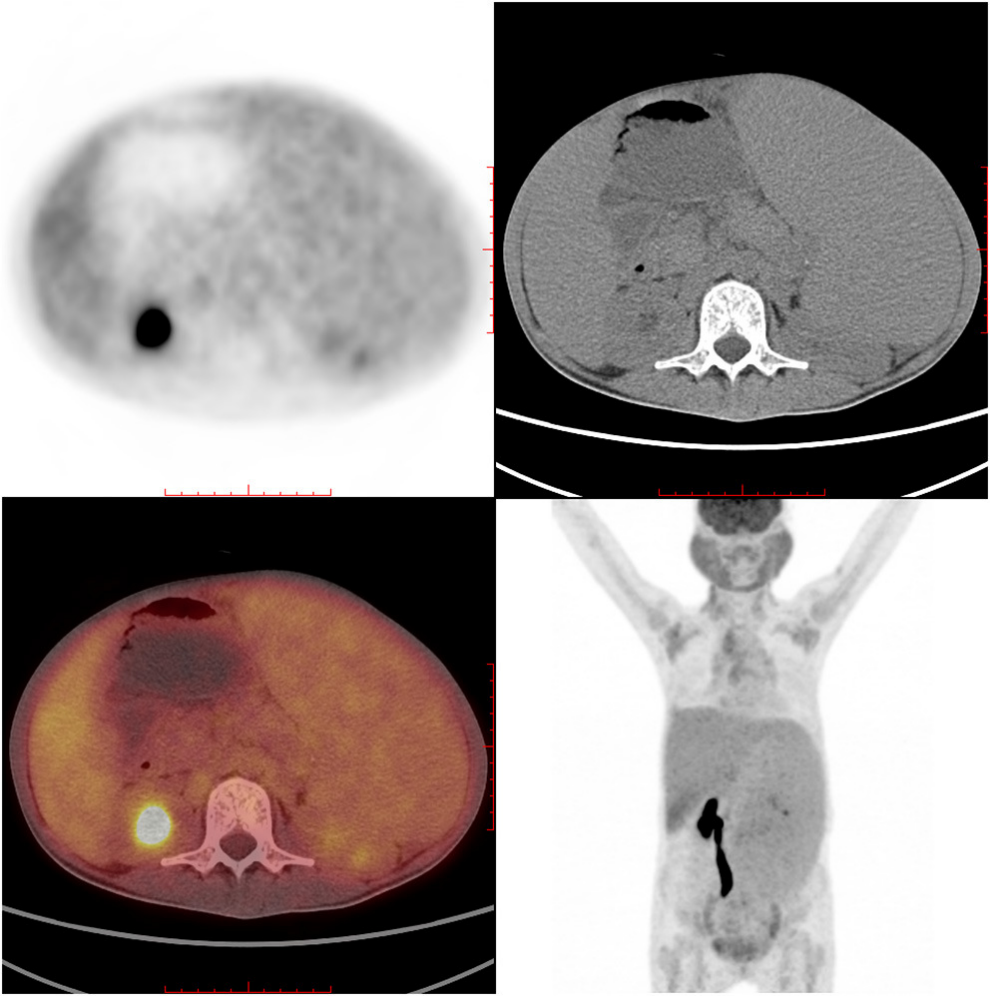
PET-CT showed high glucose intake in salivary glands, lymph nodes, liver, and spleen.

IgG4-RD was suspected. Parotid gland biopsy showed dense lymphocyte infiltration with >40% IgG4-positive plasma cells. No sign of Castleman disease or lymphoma was identified (Figure 3). An 18F-labeled fluorodeoxyglucose positron emission tomography identified high glucose intake in diffusely enlarged salivary glands, lymph nodes, liver, and spleen (Figure 4).

The unexplained leukocytosis added complexity to the clinical scenario. Blood smear revealed nucleated erythrocytes and 1% immature granulocytes. Bone marrow aspiration showed a myelodysplastic pattern with 2.5% myeloblasts. Bone marrow biopsy showed histopathological signs of fibroplastic proliferation. BCL-ABR translocation was negative. Noticeably, genetic mutation screening by polymerase chain reaction test of peripheral blood reported mutations of MPL (81.17%), IDH (45.38%), and FLT (44.9%). Primary myelofibrosis was diagnosed.

Under the diagnosis of IgG4-RD, the patient started intravenous methylprednisolone 40 mg once daily for 2 weeks. She also started oral mycophenolate mofetil 0.5 g twice daily. The enlargement of salivary glands and adenopathy alleviated substantially (Figure 2B). The level of IgG decreased to 48.71 g/l and IgG4 to 1,850 mg/dl on discharge. She continued to use oral methylprednisolone once daily for 2 weeks and then tapered the steroid to 4 mg per week. For myelofibrosis, the patient received oral thalidomide 50 mg once daily, danazol 0.2 g three time daily, and aspirin 0.1 g once daily. However, leukocytosis was refractory, and WBC increased to 1,290,000 per cubic millimeter 2 months after discharge. Ruxolitinib was considered but was inaccessible.

The patient then failed to come back to our hospital, and we tried to maintain telephone follow-ups. Adenopathy remained stable while steroid tapering. WBC levels ranged from 700,000 to 1,000,000 per cubic millimeter. Five months later, the patient was admitted to the local hospital due to fever and shortness of breath. She was diagnosed with acute monoblastic leukemia as bone marrow aspiration reported 25.5% myeloblasts. Bone marrow biopsy showed extreme myeloproliferation. Fluorescence *in situ* hybridization identified the cytogenetic feature of monosomy 7 or deletion 7q (-7/7-q). Karyotype analysis showed 46, XX, t (3:21) (q21; q22) (8) /46, XX (2). The patient received gemcitabine for 5 days but unfortunately died of septic shock in the 8th month after discharge.

## Discussion

IgG4-RD is a heterogeneous multisystem disease that presents common pathologic features but with unclear etiology. Clinical phenotypes mainly include autoimmune pancreatitis, sclerosing cholangitis, salivary and lacrimal gland enlargement, lymphadenopathy, orbital disease, periaortitis, lung involvement, tubulointerstitial nephritis, and retroperitoneal fibrosis (4). In 2019, the ACR/EULAR updated a stepwise comprehensive classification criteria of IgG4-RD, which has been validated in large cohorts (5). Our patient had typical organ involvement, including the salivary glands, lymph nodes, and kidney. No exclusion criteria were present. The classification criteria were met with total inclusion points adding up to 42. The rapid response of sialadenitis and dacryoadenitis to corticosteroid also supported IgG4-RD.

Numerous inflammatory and neoplastic processes may mimic IgG4-RD. Correlating with clinical features, differential diagnosis for this case mainly included lymphoproliferative diseases and Sjogren's syndrome. According to the biopsy of the parotid gland as well as bone morrow aspiration, there was no histological evidence of Castleman disease or lymphoma. Sjogren's syndrome is a systemic autoimmune disease causing secretory gland dysfunction that often leads to Sicca symptoms. However, it could not explain the elevated serum IgG4 concentrations. Besides, this patient was negative for anti-SSA/SSB autoantibodies, and parotid biopsy did not show focal lymphocyte infiltration either.

The co-occurrence of myelofibrosis with IgG4-RD was unique. An increased bone marrow fibrosis can occur in various benign or malignant conditions, including myeloproliferative neoplasms (MPNs), autoimmune myelofibrosis (AIMF), lymphoid neoplasms, metastatic solid tumors, chronic infections, and metabolic disorders (6). While other conditions could be excluded, it was interesting to distinguish AIMF from primary myelofibrosis (7). Vergara-Lluri et al. reported 29 patients with AIMF and described characteristic bone marrow findings, including erythroid and megakaryocytic hyperplasias, mild reticulin fibrosis, intrasinusoidal hematopoiesis, T-cell pattern in lymphoid aggregates, mild polytypic plasmacytosis, and absence of IgG4-positive plasma cells. Our case was limited because the fibrosis grade, and the presence/absence of plasma cells and their IgG4 status were not analyzed due to the quality of bone marrow specimen. However, AIMF typically followed a benign course and responded well to steroids, which contradicted with the reported case. More importantly, genetic mutation test shed light on the diagnosis of MPN. The thrombopoietin receptor, MPL, is the key cytokine receptor in MPN development, activating MPL-JAK-STAT signaling in MPN stem cells (8). Moreover, the IDH mutation of the patient indicated a high risk of AML transformation and thus predicted a worse prognosis (9). Noticeably, when the patient deteriorated 8 months after discharge, cytogenetic exams identified an adverse abnormality of monosomy 7 or deletion 7q (-7/7-q) (10). These complex genetic and cytogenetic abnormalities might be a background where two distinct disease spectrums of IgG4-RD and myelofibrosis occurred simultaneously.

For clinical physicians, it is important to realize timely when malignancy is likely to occur in IgG4-RD. Although an increased risk of solid tumors was noticed in IgG4-RD (1, 2), hematological malignancies were less reported. Data on myeloid abnormalities accompanying IgG4-RD was particularly limited, with only Guo et al. describing a case of acute lymphoblastic leukemia (11). Despite the potential pathogenetic association between IgG4-RD and myelofibrosis, it might also be a co-incidence in our unique patient. For example, the complex and contradictive observations between ANCA-associated vasculitis and IgG4-RD described the similar scenario (12, 13). This reported case suggested that MPNs may co-occur with IgG4-RD. An active search for hematological malignancies is warranted at diagnosis and during follow-up for patients who present with unexplained leukocytosis, pancytopenia, splenomegaly, or weight loss.

## Conclusion

IgG4-RD is a heterogeneous entity with diverse clinical clusters where malignancies should be carefully excluded. To our best knowledge, this is the first case with IgG4-RD accompanied by myelofibrosis that ultimately transformed into AML. Hematological malignancies, including MPNs and leukemia, should be suspected for patients who present with unexplained leukocytosis, pancytopenia, splenomegaly, or weight loss. Genetic mutation test can be helpful for diagnosis, especially when results of bone marrow aspiration and biopsy were atypical.

## Data Availability Statement

The original contributions presented in the study are included in the article/Supplementary Material, further inquiries can be directed to the corresponding author/s.

## Ethics Statement

Written informed consent was obtained from the individual for the publication of any potentially identifiable images or data included in this article.

## Author Contributions

ZL participated in the management of the patient and wrote the manuscript. SZ treated the patient and revised the manuscript. WZ, JF, ML, and XZ participated the management and follow-up of the patient. All authors contributed to the article and approved the submitted version.

## Conflict of Interest

The authors declare that the research was conducted in the absence of any commercial or financial relationships that could be construed as a potential conflict of interest.
